# What RNA World? Why a Peptide/RNA Partnership Merits Renewed Experimental Attention

**DOI:** 10.3390/life5010294

**Published:** 2015-01-23

**Authors:** Charles W. Carter

**Affiliations:** Department of Biochemistry and Biophysics, University of North Carolina at Chapel Hill, Chapel Hill, NC 27599-7260, USA; E-Mail: carter@med.unc.edu; Tel.: +1-919-966-3263; Fax: +1-919-966-2852

**Keywords:** catalysis, enzyme superfamilies, sense/antisense genetic coding, operational RNA code, tRNA acceptor stem recognition

## Abstract

We review arguments that biology emerged from a reciprocal partnership in which small ancestral oligopeptides and oligonucleotides initially both contributed rudimentary information coding and catalytic rate accelerations, and that the superior information-bearing qualities of RNA and the superior catalytic potential of proteins emerged from such complexes only with the gradual invention of the genetic code. A coherent structural basis for that scenario was articulated nearly a decade before the demonstration of catalytic RNA. Parallel hierarchical catalytic repertoires for increasingly highly conserved sequences from the two synthetase classes now increase the likelihood that they arose as translation products from opposite strands of a single gene. Sense/antisense coding affords a new bioinformatic metric for phylogenetic relationships much more distant than can be reconstructed from multiple sequence alignments of a single superfamily. Evidence for distinct coding properties in tRNA acceptor stems and anticodons, and experimental demonstration that the two synthetase family ATP binding sites can indeed be coded by opposite strands of the same gene supplement these biochemical and bioinformatic data, establishing a solid basis for key intermediates on a path from simple, stereochemically coded, reciprocally catalytic peptide/RNA complexes through the earliest peptide catalysts to contemporary aminoacyl-tRNA synthetases. That scenario documents a path to increasing complexity that obviates the need for a single polymer to act both catalytically and as an informational molecule.

## 1. Introduction

**Figure 1:**
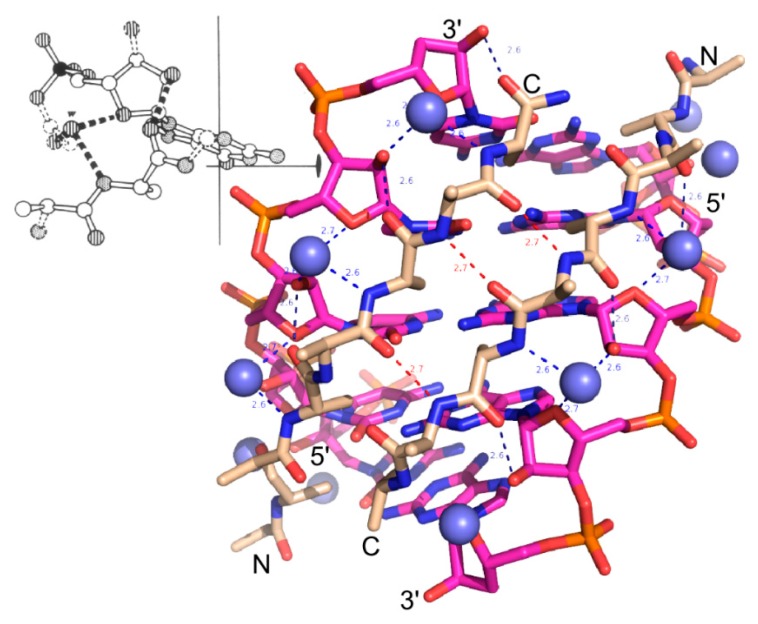
Stereochemistry of peptide-RNA conformational complementarity [1]. The minor groove in double-stranded RNA (magenta) complements the preferred right-hand twist of antiparallel β hairpin structures (wheat). Adjacent nucleotide and peptide strands are parallel (5'-3'; N-C) and the two sets are antiparallel. Van der Waals distances between peptide and nucleotide components are optimal precisely at a peptide radius for which there are exactly two amino acids per base. (**Inset**) The double-double helix is also stabilized by recurring hydrogen bonds between peptide carbonyl and the ribose 2'OH groups and between amide nitrogens and water molecules (blue spheres) between the ribose O1 and 2'OH groups. The resulting hydrogen-bonded network stabilizes a ribose orientation such that the 3'OH group is poised to serve as a nucleophile for polymerization.

**Figure 2:**
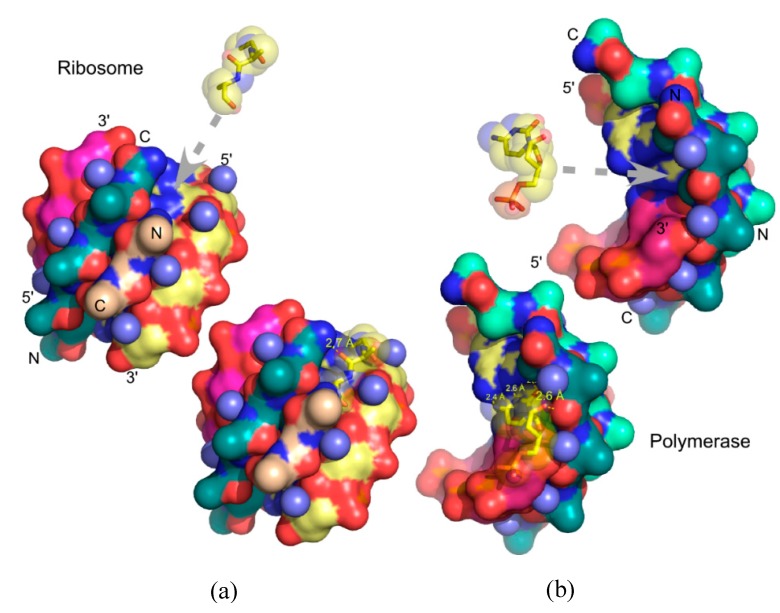
Templating and reciprocal autocatalysis proposed by Carter and Kraut. Rudimentary coding and catalytic surfaces are evident in both oligomers. Screw dislocations of peptide strands relative to nucleotide strands create pockets for the addition of new units—either a dipeptide or a nucleotide. (**a**) Addition of amino acid or dipeptide. RNA strands afford a scaffold allowing one peptide strand (teal) to serve as template and the other (wheat) as primer; (**b**) Addition of a nucleotide. Antiparallel β-structure affords a scaffold on which one RNA strand (yellow) functions as template while the other (magenta) functions as primer.

In 1974, Carter and Kraut [1] showed by model building that the range of stable twisted conformations of extended polypeptides included a double-helical configuration that precisely complements the A form RNA double-helix (Figure 1). They proposed that this complementarity, and specifically a repeating hydrogen bond between ribose 2'OH groups and outward-pointing carbonyl oxygen atoms, suggested a basis for reciprocal pre-biotic autocatalysis, in which screw dislocations between the two partners could serve, respectively, as rudimentary active-sites for catalysis of subsequent polymerization of peptides by RNA and RNA by peptides (Figure 2) [1,2]. Thus, they afford simultaneously a stereochemical coding mechanism as well as a prototypic ancestral ribosome and polymerase. This affords an unproven, but logically consistent explanation for the fact that contemporary proteins are assembled by a ribozyme and contemporary nucleic acids are made by a protein polymerase. Although the stereochemistry of this model is compelling, it has not been tested experimentally. Indeed, the odyssey sketched here back again to this model as a possible origin for subsequent biological evolution has been indirect and replete with discovery. It makes a compelling case for pursuing experimental tests of the Carter–Kraut model.

Prevailing ideas about the origin of biology [3] derive fundamentally from two notions: (i) that RNA replication according to Watson–Crick base pairing is the basis for genetic inheritance; and (ii) the necessary catalysts were initially entirely RNA-based and did not include genetically encoded proteins. The first notion, nearly a truism, is unexceptionable. However, the idea that coded peptides functioned catalytically in early stages of the origin of life directly contradicts the second central tenet of the “RNA World” scenario. Aggressive dismissal of peptides from equal partnership with RNA [4] is nevertheless surprising, given the pervasive roles played by proteins in contemporary biology, their exclusive role in polymerizing nucleic acids, and the obvious necessity of accounting for the evolutionary appearance and subsequent phenotypic selection of the genes that coded for them.

The origin of life conflates three problems—inheritance, catalysis, and coding—all of which can be viewed fundamentally as problems of emerging specificity. The basis for specific inheritance became self-evident with the discovery of base-pairing [5]. Modeling the emergence of specific transition-state recognition (catalysis) and translation (coding) poses far greater difficulty.

The nexus of these problems seems to be a lack of adequate models for the origins of genetic coding. Despite its redundancy, the universal genetic code is highly specific, and there has been no way to account for its gradual emergence by phenotypic selection from among more simply coded peptides. The absence of transitional links between earlier intermolecular interactions and the triplet code is a fundamental stumbling block that has continued to justify the questionable conclusion that a biologically sufficient set of functional RNA molecules arose by themselves, providing all informational continuity and catalysis [6] necessary to produce the code, without then leaving a trace behind in the phylogenetic record.

Reciprocal templating based on the structural complementarity of peptides and RNA proposed by Carter and Kraut affords rudimentary implementation of all necessary functionalities—coding, catalysis, and replication—by far simpler, stepwise processes. The important distinction between this scenario and the RNA World hypothesis is that the requisite specificity is low in the initial stages of the former, but unacceptably high in the latter [7]. Low specificity processes occur with greater frequency and hence are more likely to have occurred first.

As the fundamental gap in understanding the origin of life is the emergence of the genetic code, it makes sense to study how the molecular machinery developed that translates that code today: the ribonucleoprotein complexes of aminoacyl-tRNA synthetaseses with their cognate transfer RNAs. A significant unification underlying the origins of translation was the recognition that the two distinct aminoacyl-tRNA synthetase superfamilies are, very probably, fundamentally related to one another, by sense/antisense ancestry. This duality was originally proposed by Rodin and Ohno [8] in response to the recognition that the 20 amino acids were activated and transferred to cognate tRNAs by two entirely distinct superfamilies of aminoacyl-tRNA synthetases [9,10,11]. Much of our recent research has tested biochemical [12,13,14,15,16] and bioinformatics [17] predictions of that model, and examined a designed sense/antisense gene with two functional products [18,19,20].

**Figure 3:**
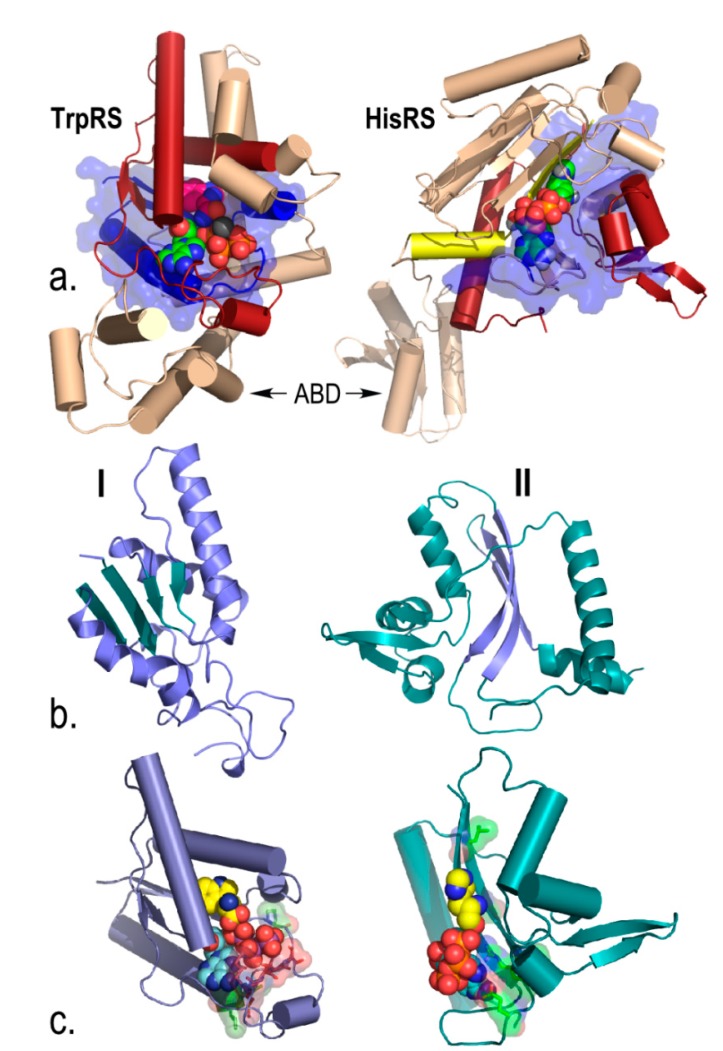
Urzymes isolated from Class I TrpRS (130 amino acids) and Class II HisRS (124 amino acids). (**a**) Monomer architectures. Both enzymes are dimeric. Monomers consist of two consensus domains, catalytic and anticodon-binding (ABD). The 46-amino acid ATP binding sites are blue and bounded by transparent surfaces; the remainder of the Urzymes are red. Catalytic domains of both also include insertions, colored amber. Active-site ligands are shown as spheres; (**b**) Secondary structures are dissimilar; Class I is a Rossmann fold with parallel β-strands; Class II is an antiparallel structure; (**c**) Amino acid (yellow) and ATP (cyan) substrates are spheres. Stick models of Class I-defining signatures PxxxxHIGH (green) and KMSKS (red) and Class II Motif 2 (green) are surrounded by transparent surfaces to reveal catalytically important interactions with ATP. The cartoons are based on crystal structures of the full-length enzymes. However, long-time MD simulations of both Urzymes in the presence of both substrates have shown that the structures shown here persist, but that in the absence of tryptophan the Class I specificity helix above the bound tryptophan re-orients, removing several key interactions involved in specific recognition [21,25,26].

“Urzymes” are quite small, highly conserved fragments of the two aminoacyl-tRNA synthetase superfamilies (Figure 3). Our biochemical studies have shown that Urzymes from both classes retain ~60% of the Gibbs energies of catalytic proficiency of fully evolved synthetases [13,14,21] and ~20% of their specificities for amino acid activation [12,20]. The catalytic power of peptides related by phylogeny to contemporary enzymes is thus far greater than was anticipated from comparison with the uncatalyzed rate of peptide bond formation, which is ~10^6^-fold slower [22,23]. This million-fold excess catalytic proficiency argues (i) that such constructs closely resemble true ancestral forms; (ii) that they are themselves highly evolved; and (iii) hence had simpler functional ancestors that might now themselves be experimentally accessible. Second, and less obvious, we showed that synthetase Urzyme coding sequences have a property—high middle-codon base-pairing—expected if these two distinct enzyme families were once encoded on opposite strands of the same ancestral gene [17], as proposed by Rodin and Ohno [8,24]. This new metric can therefore also be used to pursue the histories of ancient genes further back into the depths of time than ever before.

## 2. Experimental Section

**Figure 4:**
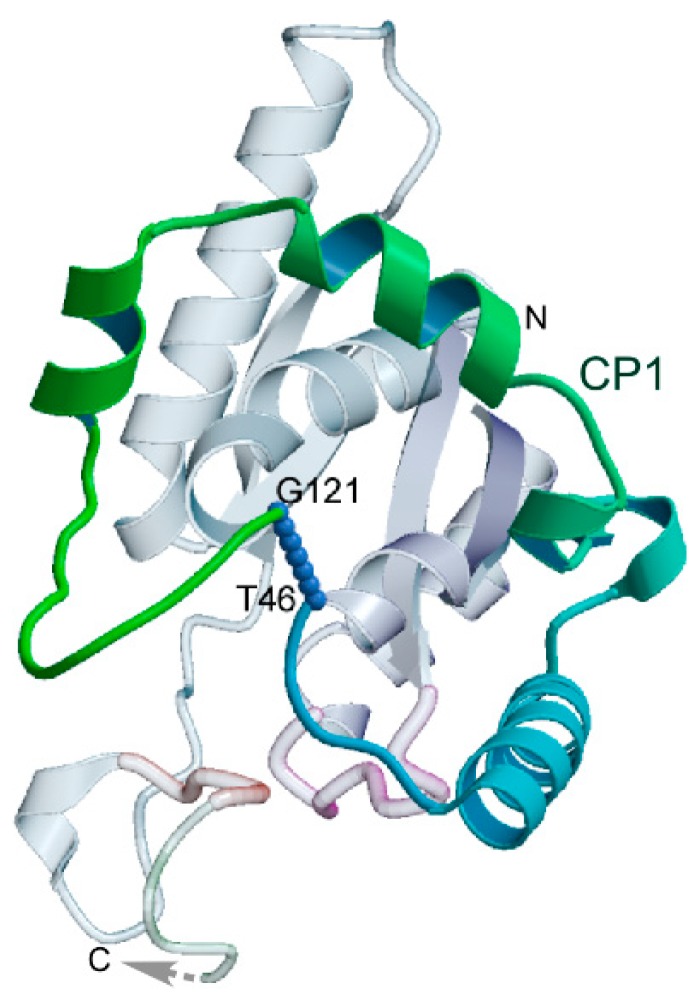
Relationship between TrpRS Urzyme and connecting peptide 1 (CP1). The alpha carbons to which the N and C termini of the insertion attach are separated by almost exactly the distance of a peptide bond (blue dots). This enables its removal by protein engineering, which was accomplished using the Rosetta design program [15,21]. The dashed arrow indicates where the anticodon-binding domain attaches (Adapted from [15]).

Urzymology entails a variety of experimental and computational studies. The underlying structural biology that enabled the production of Urzymes is that deconstruction of Class I TrpRS revealed obvious modular components, and these components relate to the fundamental structure of the Urzyme in recognizable ways. The constructs we have made included several ambitious protein engineering applications, to achieve with proteins what is much more straightforward working with RNA. A putative insertion element identified as connecting peptide 1 (CP1) by Schimmel and co-workers [27,28,29] is located such that it can be excised and replaced by a peptide bond [15,21] (Figure 4). This is true for the CP1 insertions present in all 11 of the Class I aaRS families, which vary in size from ~75 to well over 500 residues. This observation made it far simpler to contemplate the radical surgery that became so useful in Urzymology. Protein engineering showed repeatedly that disjoint active site fragments of the two earliest enzyme families can be re-joined without up to 75% of the contemporary genes to form functional catalysts. These experimental proofs-of-concept show that the reverse process, insertion, is a valid evolutionary mechanism for the growth of complexity.

Excerpting the Urzymes from both Class I and Class II aaRS presented another problem, which was that the newly exposed hydrophobic surface area led to greatly reduced solubility. This problem was initially addressed by using Rosetta [30] to reconfigure the newly exposed surface area and by renaturing TrpRS Urzymes from inclusion bodies by reducing (Guanidinium Hydrochloride) [15]. Later, all Urzymes were expressed as maltose-binding protein fusions [14,21], which brought substantial fractions of the expressed Urzymes into the soluble fraction and permitted purification on amylose beads. That protocol was also adapted for use with 46-residue peptides from a designed gene in which the ATP binding sites of Class I and II aaRS were encoded by Rosetta on opposite strands if the same gene [18,20].

**Figure 5:**
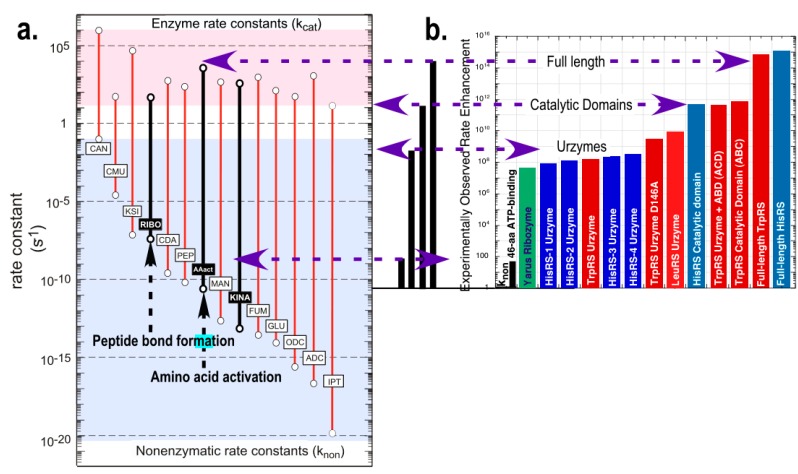
Quantitative framework in which to assess the catalytic significance of Urzymes and various other putative stages of aaRS evolution. (**a**) Rate accelerations estimated from experimental data for single substrate (red) and bi-substrate (Black, Bold) reactions adapted from [31] to include uncatalyzed and catalyzed rates of bi-substrate reactions of the ribosome [22,23], amino acid activation [32], and kinases [33]. Second-order rate constants (black bars) were converted into comparable units by multiplying by 0.002 M, which is the ATP concentration used to assay the catalysts shown in B; (**b**) Experimental rate accelerations for amino acid activation, estimated from steady-state kinetics of ^32^PPi exchange as k_cat_/K_M_ for catalysts derived from Class I and Class II aminoacyl-tRNA synthetases [14,21]. Vertical scales in (a) and (b) are the same, and the origin of the histogram in (b) is set equal to the uncatalyzed rate of amino acid activation (AAact) in (a). Red bars denote Class I Tryptophanyl- and Leucyl-tRNA synthetase constructs, blue bars denote Class II Histidyl-tRNA synthetase constructs, black vertical lines denote catalysis by 46-residue ATP binding sites, and green denotes a ribozymal catalyst [34] for a different amino acid activation reaction, included for comparison. Research presented in (a), (b) was originally published in [13]. © The American Society for Biochemistry and Molecular Biology.

Urzyme catalytic activities discussed in greater detail in Section 3 (Table 1 and Figure 5) are variable within ~100-fold. The D146A mutation of the TrpRS Urzyme ) [15] increases activity 25-fold. Thus, some wild-type active-site residues—D146 in TrpRS—have assumed specialized catalytic functions in the presence of later inclusion of catalytic domain insertions such as the Class I connecting peptide 1, CP1, and the anticodon-binding domains. We have verified this idea experimentally in some detail [25,26]. The most active aaRS Urzymes—those excerpted from LeuRS and the active-site mutant D146A of the TrpRS Urzyme—have transition-state stabilization energies that are ~60% of those of contemporary enzymes, and they therefore are about 10^−5^ times as active.

Contamination, either by wild-type or by other adventitious catalysts, poses a significant problem as an explanation of the observed catalytic activities. Remarkably, however, all the Urzymes we have examined share with their putative contemporary descendants the fact that they bind tightly enough to the aminoacyl-adenylate intermediate to produce a pre-steady state burst, whose amplitude can be used to estimate the fraction of molecules contributing to the observed signal. This means that the authenticity of the activities can be established by performing active-site titration, showing that burst sizes correspond to a major portion of the molecules present in the catalytic sample [14,21]. Other stringent tests for authenticity include showing that Urzymes have K_M_ values far from those of contemporary wild-type enzymes, and that mutant or modular variants have different steady-state kinetics. For peptides smaller than Urzymes, including the 46mers containing the ATP binding sites, active-site titration is no longer an option, but active-site mutation and steady state K_M_ values still provide evidence of authenticity [18].

**Table 1:** Catalytic rate constants for catalysts in Figure 5b.

Catalyst	k_cat_/K_M_, /s/M
Uncatalyzed	8.30 × 10^−9^
Class I SAS 46mer	2.70× 10^−7^
Class II SAS 46mer	2.90× 10^−7^
HisRS 46mer	3.1× 10^−7^
TrpRS 46mer	1.9× 10^−6^
HisRS-1 Urzyme	7.26× 10^−1^
HisRS-2 Urzyme	1.13
TrpRS Urzyme	1.36
HisRS-3 Urzyme	2.02
HisRS-4 Urzyme	2.76
TrpRS Urzyme D146A	2.65 × 10
LeuRS Urzyme	7.84 × 10
HisRS_Cat_domain	4.13 × 10^3^
Full-length TrpRS	6.49 × 10^6^
Full-length HisRS	9.96 × 10^6^

Bioinformatic procedures used in our work [17] derive from a protocol provided by J. Thornton, and have made use of Muscle [35], ProtTest [36], JModelTest [37], and PAML [38]. In addition, we introduced the middle-base pairing metric, a novel procedure for comparing superfamily genes that might be related by sense/antisense ancestry [17]. Coding sequences in multiple alignments of such genes are aligned antiparallel to one another, using three-dimensional structures to align “anchors” containing the most conserved amino acids thought to be related by sense/antisense coding. Then, the middle codon bases on each strand are examined to see if they form a base pair. The mean frequency of such base pairing in all-by-all antiparallel alignments, <MBP> (middle-base pairing), and its standard error are compared with a large number of such alignments representing the null hypothesis, and which cluster tightly around a value of 0.25.

## 3. Results and Discussion

Unavailability of activated amino acids was the most critical barrier to the emergence of protein synthesis. Thus, accelerated production of activated amino acids by 10^8^–10^9^-fold and of aminoacylated tRNA by 10^6^-fold represented by peptide catalysts like aaRS Urzymes [13] was, almost certainly, a key driving force for a dramatic stage in the evolution of the genetic code. Uncatalyzed amino acid activation estimated from the rate of reacting acetate with methyl-2,4 dinitro phenhyl phosphate [32] is 10^3^–10^4^-fold slower than uncatalyzed peptide bond formation from free amino acids [22,23]. Thus, it seems unlikely that an emergent PTC could have assembled polypeptides without a supply of activated amino acids. Indeed, in this light, amino acid activation appears to be the rate-limiting chemical step preventing the emergence of genetically coded proteins. Urzymes and especially even earlier ancestral aaRS thus almost certainly played a central role in the origin of translation. Ribosome catalysis of peptide bond formation from free amino acids has not been studied. However, the contemporary ribosome accelerates peptide bond formation from activated amino acids by only ~10^7^-fold [22], making it unlikely that primitive ribosomes gained a selective advantage much before indirect coding of catalysts like the aaRS Urzymes emerged.

Urzymology, the ability to reconstruct invariant cores from protein superfamilies and examine their experimental behavior, has transformed the study of the origin of ancestral aaRS by furnishing a hierarchical set of constructs, for both Classes, whose steady-state kinetic and specificity parameters correspond to consensus phylogenetic hierarchies (Table 1; Figure 5). Four rigorous, complementary tests prove that the observed activities arise authentically from the fragments and are not due to adventitious contamination [14,21]. Both Urzymes catalyze acylation of tRNA [13]. Reconstructed catalysts containing the most conserved and, by consensus, the most ancient 12%–20% of the contemporary genes, retain 60% of the transition state stabilization energies of contemporary enzymes *in both reactions necessary to translate the genetic code*.

An important implication is that a succession of simpler peptides ancestral to intact aaRS should exhibit substantial catalytic rate enhancements of the two chemical reactions necessary to translate the genetic code. Structural hierarchies in native aaRS of both Classes run from the native enzymes ~800 to ~400 residues, through catalytic domains of ~250 residues (synthetase catalytic domains in both classes include 80–300 residue insertion subdomains), and Urzymes of ~125 residues, to the ATP binding sites of ~46 residues (see Figure 3a; [18,19,20]). Catalytic proficiencies increase in parallel (Table 1; Figure 5b), spanning 11 orders of magnitude. The properties of these constructs sequentially and logarithmically reduce the gap between the rudimentary model advanced by Carter and Kraut and the shortest experimentally validated catalysts with recognizable phylogenetic connections to contemporary aminoacyl-tRNA synthetases. Irrespective of how closely they actually resemble ancestral catalysts, these hierarchies demonstrate that peptide-based catalysis and specificity are striking attributes of peptides far shorter than similar contemporary enzymes.

### 3.1. Urzymes are a Logarithmic Mean between the Earliest Catalysts and Contemporary aaRS

The Urzymes represented by constructs derived from Class Ic TrpRS, Class Ia LeuRS, and Class IIa HisRS have apparent second-order rate constants, ~0.1–80 /s/M (Table 1), that are ~10^5^ times slower than those of full-length aaRS and ~10^5^ times faster than those of isolated ATP binding sites, ~3 × 10^−7^ /s/M [18]. The size of Urzymes, relative to comparable contemporary enzymes, can be appreciated first by recognizing that they have four substrates: ATP, amino acid, tRNA, and PPi. Moreover, synthetase Urzymes not only activate amino acids and acylate tRNAs, they also retain the activated amino acids with high affinity. Thus, they retain three essential properties of full-length synthetases.

Contemporary enzymes the size of aaRS Urzymes exist, but they are hydrolases and isomerases that act on a single substrate; multisubstrate enzymes generally have considerably more mass [39]. The average modular molecular weight of 5000 Kd/ligand from a survey of molecular mass required per ligand bound [40] suggests a minimum molecular mass of 20 Kd for such enzymes. In fact, enzymes that bind nucleotide ligands from that survey have a mean molecular mass of 41 Kd with a standard error of the mean of 1.9 Kd. Synthetase Urzymes are smaller than such enzymes by 14 times the standard error.

Thus, Urzymes appear to be an important experimental platform from which to explore both forward [25,26] and backward [18,19] in time [41]. Moreover, they also seem to be the smallest segments that retain all three of the activities associated with faithful translation of the code [13]. Amino acid activation is necessary to drive peptide formation thermodynamically; aminoacyl-adenylate retention is a necessary precondition for enhancing amino acid specificity; and tRNA aminoacylation affords the crucial link that enabled codon-dependent amino acid assembly.

From their intermediate states, aaRS Urzymes afford in addition a crucial baseline for examining how they evolved to assume their contemporary size and specificity. They function in this case as molecular knockouts, establishing a general, quantitative experimental reference for measuring theenergetic coupling between more recently accumulated domains. Perhaps most unexpected of the observations we have made is that all functionality present in contemporary enzymes, but absent from Urzymes, arises exclusively from allosteric energy coupling between more recently accumulated domains (see below, Section 3.7; [12,25,26]).

### 3.2. Urzyme Specificities are Consistent with Implementing Statistical Peptide Ensembles

**Figure 6:**
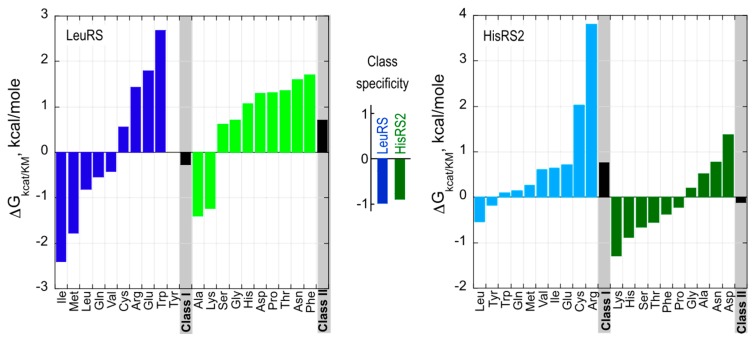
Amino acid specificity spectra of Class I LeuRS and Class II HisRS2 Urzymes. The net free energy for specificity of Class I and II Urzymes for homologous *vs.* heterologous substrates,* i.e.*, ΔG(k_cat_/K_M_)_ref_ – ΔG(k_cat_/K_M_)_amino acid (i)_, where ref is the cognate amino acid, is approximately 1 kcal/mole for both Class I and II Urzymes (center). Class I amino acids are colored blue; Class II amino acids are colored green. Bold colors denote substrates from the homologous Class; pastel colors denote heterologous substrates.

The small sample of two aaRS Urzymes examined thus far retains ~20% of the Gibbs energy by which the full length enzymes achieve specific amino acid recognition ([20]; Figure 6). Urzymes derived from the two Classes favor amino acid substrates from their own class by ~1 kcal/mol. These unprecedented experimental data are the first to frame in quantitative terms the suggestion of Woese that the first coded peptides were probably statistical ensembles [42,43] with homologous sequences, and varying ranges of functionality. That situation highlights a key stage in the evolutionary development of specificity required for any acceptable scenario describing continuous emergence of complexity from randomness. In this light, it seems far more likely that the complexity of nucleic acids and proteins grew together than it is that one polymer emerged first without the aid of the other.

### 3.3. Urzymology in the Context of Similar Analyses of Ribosome Evolution

Williams [41,44,45] has directed a similar effort to our own that has been devoted to reducing the size of the ribosomal 23S RNA containing the peptidyl-transferase center. Analysis of the thermodynamics of catalyzed and uncatalyzed peptide bond synthesis by that catalyst [22,23] shows that it is substantially more primitive, even in fully evolved ribosomes, than the active sites of the aaRS. The reason for this is that the uncatalyzed rate of peptide bond synthesis from activated amino acids is itself so much faster than that of amino acid activation. Nonetheless, the apparent evolution of 23S RNA appears to follow stages of accretion that are reminiscent of those we have described for the two aaRS Classes, and the simplest potential catalyst identified by that group is, proportionately, the same size as the aaRS Urzymes. That RNA fragment can be shown to catalyze a model peptide bond synthetic reaction, although despite some effort, the group has not successfully shown catalytic turnover. Nor have they measured steady-state kinetic parameters. This may be because the simplest functional peptidyl-transferase center requires ribosomal proteins L2 and L3 [46]; (H. Noller, personal communication), illustrating how the ideal of an RNA World may have stalled otherwise productive lines of investigation.

### 3.4. Sense/Antisense Ancestry Furnishes Key Links Backward to Simpler Genetics

We have now extensively validated Rodin and Ohno’s hypothesis that Class I and II aaRS descended from opposite strands of the same gene [20]. That validation unifies Class I and Class II aminoacyl-tRNA synthetase superfamilies that heretofore were considered distinct. This unification is unlike the nodes from any previous ancestral reconstruction because it implies that the unique information in a gene can have two equally valid interpretations and lead to descent of two distinct, but complementary superfamilies. We have argued elsewhere [12,16,20] that the ancestral synthetases also gave rise to numerous other contemporary superfamilies—Class I synthetases to the Rossmannoid group of proteins, and Class II synthetases to the Actin-HSP 70 group. These two meta-families comprise a substantial fraction of the contemporary proteome [47,48,49].

Middle-base complementarity of genes descended from opposite strands of an ancestral gene increases as reconstructed nodes approach the roots of the two respective trees, extending phylogenetics back well beyond its present limits. Sense/antisense ancestry thus affords a new phylogenetic and bioinformatic metric, opening a path to discriminate between alternative processes by which the aminoacyl-tRNA synthetases came to use only a single strand of modern genes, and how they radiated to new species that enlarged a partial genetic code [17]. The middle-base pairing metric may project back in time to quite short peptides and is a potential source of useful data on events well beyond that accessible via conventional phylogenetics, implying that some of the earliest coded peptides might be identifiable from their coding complementarity.

### 3.5. Links Connecting the Sense/Antisense 46mer Gene to the Carter and Kraut Model

Two recent threads have substantively improved the credibility of the Carter and Kraut proposal [12,20].

#### 3.5.1. Amino Acid Activation Is Accelerated by 46-Residue ATP Binding Sites from Both aaRS Classes

First, we have characterized the functionality of segments roughly a third the length of the TrpRS Urzyme. These correspond to the ATP binding sites of the contemporary synthetases (Figure 3a). It seems implausible that such small polypeptides would stably fold, given that they are not coordinated to a metal ion and have not been selected for stability. Yet there is quite good precedent for such activities. The Class I 46mer is a distant homolog of ~50 residue peptides excerpted from F1 ATPase, adenylate kinase, and DNA polymerase I by Mildvan [50,51,52,53,54]. Those studies demonstrated both ATP dependent folding and high affinity ATP binding. Class I and II 46mers also bind ATP and catalyze cognate amino acid activation ~400-fold. We have designed and characterized a *bona fide* sense/antisense gene, using Rosetta to decorate fixed backbones of the Class I and II 46mers using amino acids with matched codon-anticodon pairs. Both gene products from that gene have comparable catalytic activities for amino acid activation by ATP that depend significantly on time, the amino acid concentration, and the peptide concentration [18]. These activities are greatly reduced by active-site mutations to the second histidine in the Class I HIGH sequence and the catalytic arginine in motif 2 of Class II, proving in principle that both strands of the unique genetic information in a gene can have valid, functional interpretations. Combined with the biochemical analysis of Class I and II Urzymes and the bioinformatic evidence for sense/antisense ancestry, these results show beyond reasonable doubt that the ancestors of two aminoacyl-tRNA synthetase families that translate the genetic code arose as complementary strands of the same gene, validating the Rodin-Ohno hypothesis [8].

**Figure 7:**
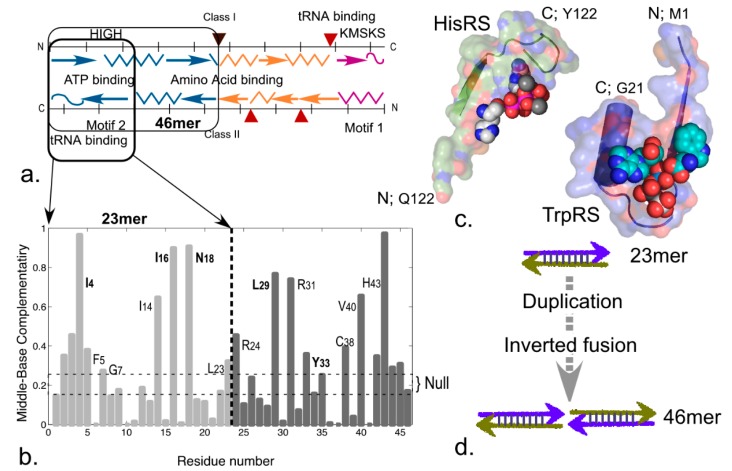
Evidence for an inverted repeat in coding for Class I ATP binding sites. (**a**) Schematic of functional divisions in the Class I and II Urzymes, showing parallel localization of ATP, amino acid, and tRNA binding sites; (**b**) Coding sequences for the 46 residue segment have significantly elevated middle-base pairing (0.28 ± 0.0005 *vs*. 0.25 ± 0.0004) when aligned antisense to each other. Frequencies of middle-base pairing between residues 4 and 43 together with 16, 18 and 29, 31 and other pairwise comparisons along the gene are evidence of coding by an ancestral palindrome; (**c**) Structures corresponding to the sense/antisense 23mer containing Class I PxxxHIGH and Class II Motif 2 binding determinants; (**d**) Putative evolution of the 46mer gene via formation of an inverted repeat. Such a gene would have been stable either as a duplex or as an RNA hairpin.

An interesting footnote is that coding sequences for the 46-residue ATP binding site of TrpRS (*i.e*., the TrpRS 46mer) exhibit significantly elevated mean middle codon base pairing in multiple antisense alignments. Middle bases of the second half of this segment have significantly elevated complementarity to the middle bases in the first half, exhibiting evidence for coding by a palindromic RNA sequence and hence by a hairpin (Figure 7a,b). Such ancestry introduces an even simpler, 23 amino acid precursor to the ATP binding site of both aaRS superfamilies. Remarkably, the major Class I and II ATP binding determinants in aaRS reside at the *N*-terminus of the Class I 46mer and at the C-terminus of the Class II 46mer. Thus both are retained in corresponding, complementary halves of the 46mer gene encoded by the same half of the sense/antisense gene, hence would be retained in the 23-mers (Figure 7c). Thus, the 46mers might themselves have arisen spontaneously from a simpler 23-residue sense/antisense gene by formation and subsequent evolution of an inverted repeat (Figure 7d).

#### 3.5.2. tRNA Anticodon and Acceptor Stem Bases Form Complementary, Non-Overlapping Codes for the 20 Amino Acids

**Figure 8:**
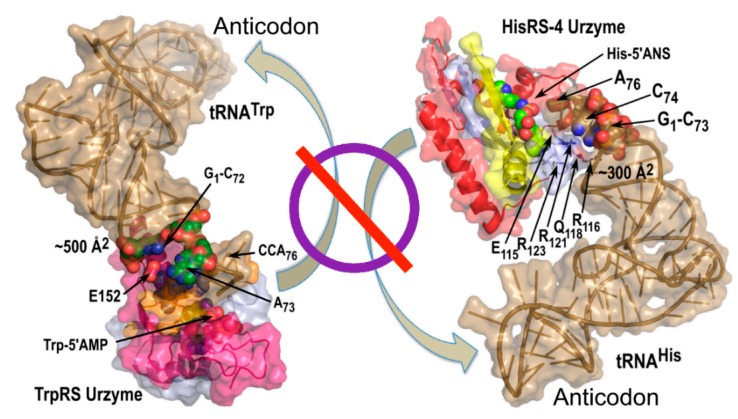
Urzyme size precludes tRNA anticodon recognition. Urzyme interactions include binding determinants for the tRNA acceptor stem, but cannot interact with the anticodon.

Motivated by our demonstration that aaRS Urzymes cannot interact with the tRNA anticodon (Figure 8) and the proposal [55] that an operational code in the acceptor stem preceded formation of the canonical genetic code, we investigated the unique coding properties of these two regions in tRNAs. We used two bits (pyrimidine *vs*. purine; number of possible hydrogen bonds in a base pair) to represent the information embedded in each base of the anticodon and acceptor-stem coding regions of tRNAs. This binary coding information for each of the 20 canonical amino acids was used to train regression models for amino acid properties, testing the models against properties of two non-canonical amino acids—selenocysteine and pyrrolysine—outside the training set [56].

Anticodon bases form a complete code for the hydrophobicities of the 20 amino acids, represented by their free energies of transfer from water to cyclohexane. Categorical variables (e.g., aromatic, basic, carboxylate, amide, aliphatic) are also completely specified by anticodon bases. However, surprisingly, acceptor stem bases form a complete code for the size of the canonical amino acid side chains, represented by mass and/or their free energies of transfer from vapor to cyclohexane. Coefficients of this model predict the sizes of both selenocysteine and pyrrolysine outside the training set within 8%. In addition, the acceptor stem uniquely predicts whether a side chain is branched at the β-carbon atom and whether or not it has a carboxylate sidechain. Thus, the coding properties in the acceptor stem have little overlap with those of the anticodon; both specify all 20 amino acids via distinct properties, basically size and hydrophobicity. The possible significance of these observations is discussed in the next section.

### 3.6. tRNA Acceptor-Stem Coding Preserves Peptide RNA Interactions of the Carter and Kraut Model

**Figure 9:**
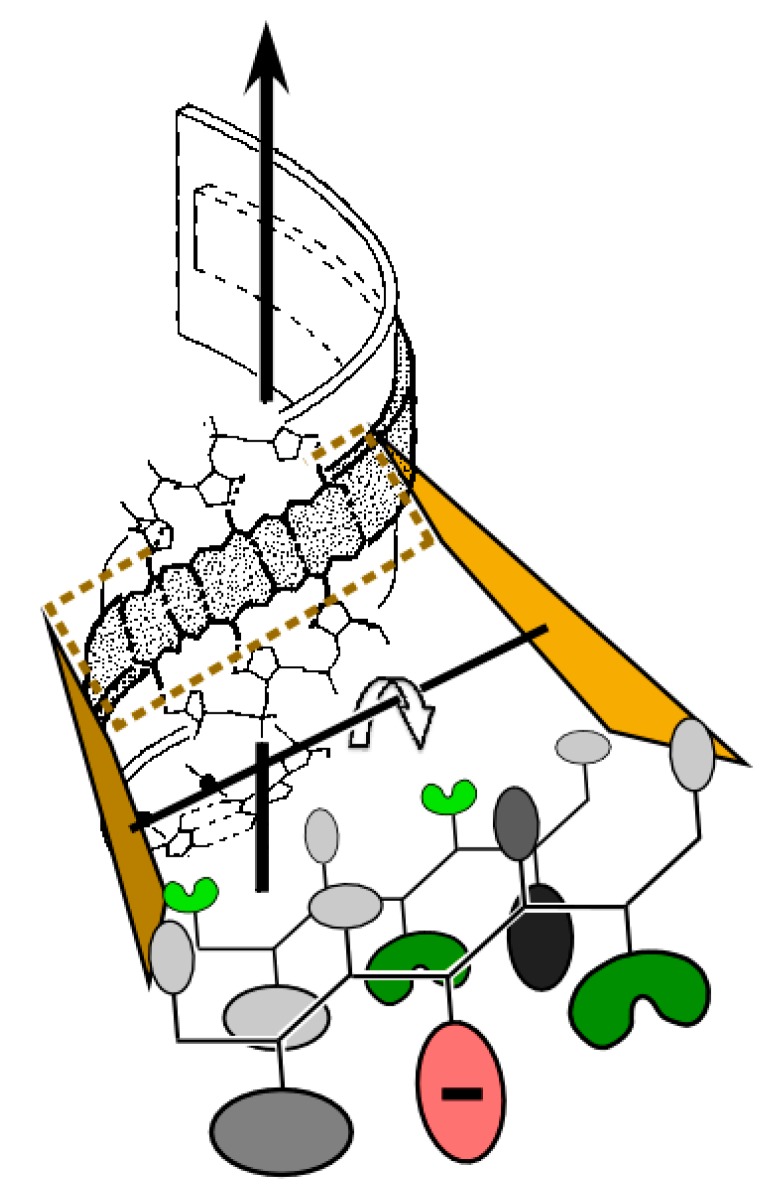
Possible relevance of mass, β-branching, and carboxylates to the operational RNA code (adapted from [56]). For ancient β-hairpins to interact with double-stranded RNA as envisioned by Carter and Kraut [1], large side chains would necessarily have faced away from the RNA minor groove. β-branched side chains on either face (re-entrant angles; green; threonine, valine on the inward face; isoleucine on the outer face) enhance β-structure formation. Carboxylate side chains in outward facing positions (red) could enhance solubility [58,59] and coordinate catalytic divalent metals, either for catalysis or to protect against RNA degradation [60].

Binding pockets of the Carter and Kraut model in Figure 2 establish symmetry between the mechanism for choosing incoming amino acid and nucleotide precursors. Incoming inward-facing amino acids of the appropriate chirality are determined chiefly by the templating peptide strand and the base of the corresponding polynucleotide strand. The proposal of Carter & Kraut thus actually implements a rudimentary sense/antisense coding in which each base in an RNA duplex codes for two amino acids, and *vice versa* each dipeptide specifies a corresponding base (Figure 1 and Figure 9). Functionalities emerging from such a primitive coding system would tend to persist and lend a selective advantage to any successive genetic coding that would preserve the ability of peptides to interact with RNA in this fashion. It is within the realm of possibility that this stereochemical coding might generate peptides (and corresponding RNA “genes”) as long and functional as the 23mer system illustrated in Figure 7. Furthermore, such a gene would have the length of a tRNA gene (~72 bases). Such an evolutionary intermediate might be expected also to preserve sense/antisense coding, consistent with the vestigial traces of such coding in the contemporary aaRS genes.

Figure 9 illustrates aspects of the Carter and Kraut model consistent with acceptor stem base coding. Large amino acid side chains at inward-facing positions would seriously disrupt peptide–RNA interactions in three ways. Displacing the antiparallel β-structure to higher radii would (i) eliminate the synchronous periodicity of dipeptides and bases; (ii) break the peptide-sugar phosphate hydrogen bonds; and (iii) break Van der Waals interactions between other inward-pointing side chains and the RNA bases. Accepter stem coding on the basis of amino acid size therefore appears central to preserving such interactions. β-branched side chains are preferentially observed in extended β-structure in contemporary proteins [57]. Selectively identifying such side chains would have the advantage of enforcing extended secondary structures, also preserving peptide–RNA interactions by a complementary constraint.

Carboxylate side chains are a curiosity. However, they could have had three different functional roles. There are multiple kinds of evidence that carboxylate side chains uniquely increase solubility, which could have been a limitation of peptides, especially before the advent of (molten) globular tertiary structures [58,59]. Alternately, carboxylate side chains may have begun to coordinate divalent metals during the earliest stages of indirect genetic coding. Carboxylate groups are the dominant ligand for Mg^2+^ ions in contemporary proteins [61]. Moreover, Mg^2+^ ions are now the dominant divalent metals in transferases and ligases [62], which are the most important catalysts related to nucleic acid metabolism. Finally, coordination of Mg^2+^ ions also has been cited as potentially crucial for limiting metal-catalyzed hydrolysis of RNA [63], and even been suggested as crucial for the emergence of stable oligonucleotides [60]. tRNA acceptor stem coding is therefore consistent with having served as an intermediate genetic coding strategy connecting the crude stereochemical coding proposed by Carter and Kraut to a regime of indirect acceptor-stem based coding [64] and ultimately to the canonical genetic code.

### 3.7. A Coherent Scenario Links the Carter & Kraut Model to Contemporary aaRS

**Figure 10:**
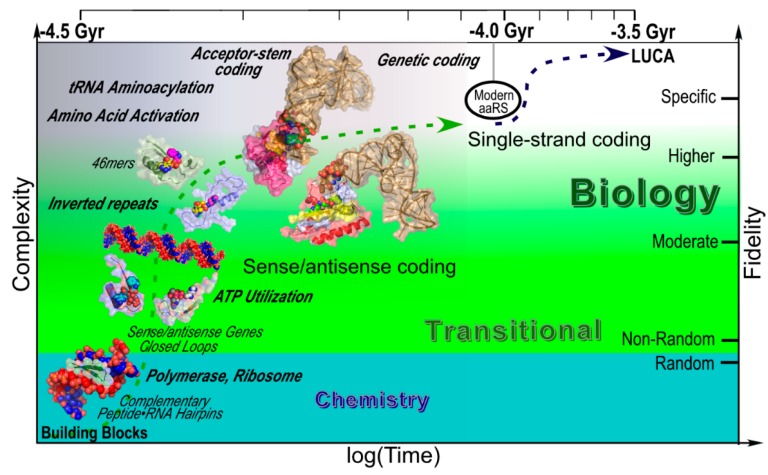
Detailed scenario for the emergence of peptide and RNA polymers and steps toward the creation of the genetic code. Approximate time intervals run from the formation of liquid water on the earth to the appearance of the last universal common ancestor (LUCA) and specifically plot stages in the evolution of the aaRS that we have documented either by model building (Figure 1 and Figure 2) or experiments (Figure 5), and which therefore are arguable intermediates that do not require prior evolution of human technology. Major biochemical events are in italicized bold face. Sense/antisense coding appeared with the first reciprocally autocatalytic peptide–RNA complex and persisted until the evolution of aminoacyl-tRNA synthetases, tRNAs, and ribosomes enabled higher-specificity genetic coding.

Figure 10 summarizes a scenario for the origins of translation and the contemporary genetic code from an ensemble of peptide–RNA complexes (Figure 1 and Figure 2). This scenario makes several assumptions. Because reciprocal autocatalysis enables the transition from simplicity to complexity, these assumptions are far more limited than those necessary to produce a population of functional polymers of only one type. The most significant assumption is that a source of chemical free energy, perhaps polyphosphate [65,66], could drive the earliest dehydration reactions necessary for monomers to oligomerize and eventually, in the same time frame, for synthesis of nucleotide triphosphates (NTPs). Among the virtues of this scenario are that it is built from pieces that have been demonstrated, often by both model building and experimental construction and assays, and that all but the earliest of the postulated molecular species have strong phylogenetic support because they derive successively from the most highly conserved amino acid sequences in contemporary aaRS.

We envision a prolonged period of chemical evolution during which amino acid and nucleic acid monomers began to assemble into covalent complexes involving structurally complementary oligonucleotides and dipeptides. Reactions accelerated in this stage would have included peptide and oligonucleotide synthesis and ligation, whose specificity would have been limited to base-pairing and a rough stereochemical coding between the two types of polymers that preferred the addition of new monomers in ways that stabilized the peptide–RNA double-double helical complex (Figure 1). Ligation activities may have been important in allowing such complexes to grow in length, perhaps to the size of the putative 23-amino acid sense/antisense gene that produced the first binding sites for nucleotide triphosphates. At this point, Class I and II 23mers may both have mobilized NTPs for biosynthetic purposes.

An important putative transformation converted the 23mer gene by ligating together an inverted repeat to form the 46mers that we have now demonstrated have significant ability to activate amino acids (and perhaps other carboxylate and alcoholic groups [67]). At approximately this time, the two polymers in the founding peptide–RNA complexes illustrated in Figure 1 began to assume specialized functions that exhibited their intrinsic selective advantages and so have persisted to contemporary biology. Some of the peptides increasingly specialized as nucleic acid polymerases, accounting for the total absence of ribozymal polymerases in even the most secluded nooks of biology that have so far been explored. Others acylated double-stranded RNA and led to functions now associated with aminoacyl-tRNA synthetases and the tRNA acceptor stem. Double-stranded RNA retained its roles of priming and templating replication [68,69] and elaborated its role as a general purpose peptidyl transferase in assembling proteins, to become the large ribosomal subunit. Single-stranded RNA began increasingly to assume a dominant templating role that evolved toward a role now recognized as messenger RNA, introducing the possibility of indirect, genetic coding by aminoacylated double-stranded RNAs.

Wächtershäuser reviews the origins of intermediary metabolism elsewhere in this volume [70]. Several aspects of the scenario in Figure 10 merit attention in the context of his discussion. Foremost of these is that we see little conflict between events posited in our scenario and those described by Wächtershäuser and others [71] concerning intermediary metabolism. A central detail in Figure 10 is the early appearance of peptides that could bind and exploit ATP and, by implication, other NTPs. By drilling down from successively simpler and more highly conserved segments of the two aaRS superfamilies, we arrive at a very simple peptide with evident functionality crucial to harnessing a source of chemical free energy to biological processes. Further, as we have proposed before [12,20], these two archetypal ATP binding motifs are today distributed widely in many protein metafamilies. Catalysis by members of the Rossmannoid metafamily spans a substantial proportion of intermediary metabolism (*i.e*., dehydrogenases, amino acid, nucleotide biosynthesis, and catabolism).

We make no proposal regarding the origin of compartmentation [72], except to note that packaging such units as we describe in Figure 10 likely would have afforded a preferential environment for much of the evolutionary growth of translation systems. In particular, it appears likely that new modules present in the Class I and II Urzymes likely began to appear and to function *in trans*. Accretion of a pyrophosphate binding site (KMSKS) in ancestors to Class I Urzymes likely led also to the emergence of a dimer interface (Motif 1) in ancestors of Class II Urzymes. Addition of the segment between the ATP binding site and the PPi binding/dimer interface segments appears to have enabled creation of amino acid binding sites, giving rise eventually to increasingly specific amino acid activating enzymes. Growth of that central segment appears always to have enhanced amino acid specificity, and eventually produced the editing domains in synthetases activating stereochemically similar amino acids. We cannot speculate on which stage in Figure 10 involved the earliest molecular species that could accelerate acylation of tRNA acceptor stems because the acceptor stem binding determinants in Class I and II Urzymes are associated with the C-termini and hence are associated with different modules in the two Classes—the 46mer fragment in Class II; the KMSKS segment in Class I. Thus, it is possible that the earliest catalysts of aminoacylation were actually from Class II.

In any case, the Urzymes appear to be the earliest of the constructs we have studied that can be demonstrated to retain all three of the functions required for specific aminoacylation: amino acid activation, pre-steady state bursts that mean retention of the activated aminoacyl-adenylate, and tRNA acylation itself. In that sense, they are an important turning point in synthetase evolution. Urzymes already exhibit significant beginnings of amino acid specificity (Figure 6 and Figure 8). They are therefore poised to initiate a last phase necessary to produce the universal genetic code.

The amino acid specificity of contemporary aaRS poses a significant challenge, because the contemporary enzymes use long-range or allosteric interactions to enforce the requisite specificity [25,26,73,74,75,76]. In a well-characterized example, interaction between the anticodon-binding domain and an annular insert to the catalytic domain (connecting peptide 1; CP1) contributes 5 kcal/mol to the specific recognition of tryptophan and rejection of tyrosine [25,26]. Furthermore, addition to the TrpRS Urzyme of either the connecting peptide 1 (CP1) or the anticodon-binding domain individually actually degrades the specificity of the resulting putative intermediate constructs. The challenge is therefore to understand how these allosteric interactions evolved by assimilation of new, interacting modules without at the same time eliminating the inherent specificity of the Urzyme. Two possible mechanisms might resolve this paradox, for example, for Class I aaRS. Either of the two domains may have begun to function *in trans*, as suggested above for the smaller modules that completed the assembly of Urzymes. Alternately, the anticodon binding domains may have joined to the Urzyme to provide a selective advantage we have not yet tested—enhancing specificity for tRNA. That scenario would enhance the likelihood that the CP1 domain was distributed throughout the Class I superfamily by a mechanism involving retrotransposition (see, for example, Figure 5 of [15]). Transfer RNA is notably closely associated with retrotransposition, serving to prime the reverse transcription of many transposons [77], and so may have played a role in distributing essentially the same module rapidly to the population of synthetases that were already functioning together with anticodon-binding domains.

### 3.8. The Carter and Kraut Model Makes More Powerful, Successful Predictions than the RNA World

Criteria for belief in scientific hypotheses began to be understood with the theorem of Thomas Bayes [78] and evolved continuously through the work of Karl Popper [79]. Hypotheses afford the basis for predictions, and successful predictions reinforce belief. Michael Yarus has articulated the case for the RNA World hypothesis in just these terms [80], affording a basis for comparing that hypothesis with the alternative one favored here.

#### 3.8.1. Predictions Arising from the RNA World Hypothesis Are Closely-Related and Self-Fulfilling

Yarus points out, appropriately, that the existence of a pentanucleotide ribozyme capable of acylating a complementary tetranucleotide “substrate” [81,82] increases the Bayesian posterior probability of the RNA World hypothesis. His reasoning is that if life as we know it was preceded by life implemented entirely by RNA molecules, then ribozymes that catalyze acyl-transfer from activated intermediates should exist. Elsewhere [83], Yarus summarizes instances of the same argument including, from his own work, the identification of oligonucleotides that recognize specific amino acids and in which are embedded either codons or anticodons for those particular amino acids [84], a ribozyme that activates amino acids [34], and RNA aptamers with high affinity for a bi-substrate analog of peptidyl transfer containing an invariant octanucleotide that is present near the ribosomal peptidyl transferase site in 23S RNA [85,86]. Another such aptamer acylates tRNA with activated amino acids [87]. The centerpiece of such arguments, however, is evidence that ribozymes can be selected and evolved with the capability of sequence-specific RNA-dependent RNA synthesis [3,88,89,90,91,92,93]. A particularly instructive example of such aptamers is one that faithfully assembles a mirror image of itself [88], thereby provisionally escaping the problem of product inhibition in RNA replication.

In a narrow sense, this restricted class of predictions has fared well in the eyes of RNA World proponents [4]; RNA aptamers with many biological catalytic activities have now been selected, and cited as fulfilling predictions of the RNA World hypothesis. In a broader, more meaningful sense, their significance is questionable because they are unrelated to any phylogenetic evidence. They are “biological” catalysts only in the indirect sense of having been produced with advanced and powerful human technologies. Indeed, it is possible [7] that the fastest evolutionary route to such catalysts is first to evolve human life.

A more exacting set of predictions reference biology. Most impressive in this category is the evidence that the ribosomal peptidyl-transferase appears to be a ribozyme [46,94,95]. That prediction is, of course, essentially accurate. However, it is exactly canceled by failure of the corresponding prediction that RNA polymerases should contain traces of ribozymes, which is starkly invalid. Very few biological RNA lineages can be linked to catalytic functions in an RNA world. One biological RNA molecule that does qualify as evidence for RNA World ancestry, however, is the T-box riboswitch [96,97], which can both recognize a specific tRNA molecule and discriminate between its acylated and unacylated forms. The T-box stands as really the only well-characterized vestige in biology, other than 23S RNA, of a possible RNA World.

#### 3.8.2. The Carter and Kraut Hypothesis Correctly Predicts Novel, Unexpected Aspects of Biology

In contrast, the Carter & Kraut peptide–RNA origin of life makes a range of truly predictive statements about replication, catalysis, specificity, and coding in biology, beginning with the correct predictions arising from Figure 2. Not only does RNA assemble proteins, but RNA itself is assembled exclusively by proteins in contemporary biology. The symmetries and structures in the Carter & Kraut model unexpectedly predict several other aspects of contemporary biology. Foremost among these is the unification of extensive portions of the contemporary proteome afforded by the sense/antisense ancestry of the two aminoacyl-tRNA synthetase classes. Continuity of the stereochemical coding of one peptide strand in the presence of another peptide strand and double-stranded RNA implies that the first indirectly coded proteins would be related to opposite strands of double-stranded RNA. In turn, that intermediate period of molecular evolution associated the genesis of the genetic code with protein synthesis machinery that read both strands of double-stranded RNA as messages. The resulting sense/antisense ancestry can still be detected in coding of contemporary aaRS [17]. An associated prediction is that the two aaRS classes would exhibit parallel structural and catalytic hierarchies (Figure 5) and, importantly, that successively less complex modular components in both Classes would retain appropriate catalytic activities, accounting for continuous selective advantage.

A second unexpected prediction is that the initial indirect coding apparatus associated with the tRNA acceptor stem bases would be adapted to preserving the secondary structures of peptides that could interact with double-stranded RNA (Figure 9; [56]). It is relevant here that amino acid hydrophobicity, long recognized as the dominant physical property of amino acids for protein folding [98,99], may have been less important during initial stages of genetic code development and not have become essential until the advent of the tRNA anticodon stem loop. The complementary coding properties of the acceptor stem and anticodon specifically imply an intermediate developmental stage in the evolution of the genetic code that previously was identified from the dual modularity of both tRNAs and aaRS [55].

Finally, the Carter & Kraut model makes testable predictions that have not yet been observed. We have shown that relatively short peptides can exhibit sophisticated catalytic properties, sketching in Figure 5 the catalytic properties of three, successively shorter sets of peptides representing increasingly highly conserved sequences from contemporary aaRS, and that they exhibit substantial catalytic activities. We recognize and will test the prediction that the 23-residue peptides bearing minimal ATP binding sites shown in Figure 7c should have both ATP-dependent conformational changes and should bind ATP.

Connecting these phylogenetically recognizable peptides to the Carter & Kraut model, however, requires at least three new experimental approaches. First, the polymerase activities of complexes homologous to those depicted in Figure 2 must be demonstrated. The work of Turk [81,82] suggests that such experiments can be made to work. Notably, however, template-directed polymerization of activated monomers to the appropriate polymer class is qualitatively distinct from the demonstrated acyl-transfer chemistry of that ribozyme and the amino acid activating ribozyme [34]. Second, reciprocal peptide–RNA polymerizing systems must be shown to elaborate polymers of sufficient length to produce tRNAs and peptides of the length that can begin to be coded by RNA messages the length of tRNAs. Finally, the structural chemistry by which tRNA acceptor stem coding can specify indirect coding of peptides in accordance with a “messenger” RNA must be demonstrated.

## 4. Conclusions

The structural biology of aminoacyl-tRNA synthetases (aaRS) furnishes a platform from which to examine experimentally the steps by which pre-biological chemistry gave rise to the universal genetic code, thereby creating genetics. A key stage in the process was likely driven by “Urzymes,” which are models we developed to represent the core catalysts embedded within two distinct, contemporary aaRS superfamilies. aaRS Urzymes contain only ~15% of the total mass of the largest synthetases. They retain ~60% of their catalytic proficiency [13], but <20% of their specificity [20]. These properties match those necessary to produce statistical ensembles of functional peptides, as proposed by Woese. The two distinct classes of aaRS that translate the code today were formerly considered to have arisen independently. We used Urzymes to show that, rather than arising independently, the two classes probably descended from opposite strands of the same ancestral gene [17], as proposed by Rodin and Ohno [8]. Our group has ventured both backward in time [12,20], investigating likely precursors of Urzymes, and forward in time, investigating how Urzymes subsequently developed epistatic mechanisms [25,26] that increased specificity, enabling the evolution of the universal genetic code. As Urzymes cannot recognize the anticodon stem-loop, it is likely that the acceptor stem code preceded the canonical genetic code. The acceptor stem code favors the capacity of polypeptide sequences to interact with double-stranded RNA. We link these numerous biochemical, phylogenetic, and structural observations to the Carter & Kraut structural model to form a credible, testable alternative to the RNA World Hypothesis for the origin of translation and the genetic code. This work does not presuppose an “RNA world,” which we feel is based on the wrong assumptions. Rather, comparison of predictions based on the two hypotheses indicate that a peptide/RNA world is substantially more predictive, and hence a more credible and probable alternative to the prevailing idea that life originated from a single polymer with both catalytic and informational functions.

## References

[1] Carter C.W., Kraut J. (1974). A Proposed Model for Interaction of Polypeptides with RNA. Proc. Natl. Acad. Sci. USA.

[2] Carter C.W. (1975). Cradles for Molecular Evolution. New Scientist.

[3] Joyce G., Orgel L.E., Gesteland R.F., Cech T.R., Atkins J. (2006). Progress Toward Understanding the Origin of the RNA World. The RNA World.

[4] Akst J. (2014). RNA World 2.0. The Scientist.

[5] Watson J.D., Crick F.H.C. (1953). A Structure for Deoxyribose Nucleic Acid. Nature.

[6] Gilbert W. (1986). The RNA World. Nature.

[7] Koonin E.V. (2011). The Logic of Chance: The Nature and Origin of Biological Evolution.

[8] Rodin S.N., Ohno S. (1995). Two Types of Aminoacyl-tRNA Synthetases Could be Originally Encoded by Complementary Strands of the Same Nucleic Acid. Orig. Life Evol. Biosph..

[9] Eriani G., Delarue M., Poch O., Gangloff J., Moras D. (1990). Partition of tRNA Synthetases into Two Classes Based on Mutually Exclusive Sets of Sequence Motifs. Nature.

[10] Cusack S., Berthet-Colominas C., Härtlein M., Nassar N., Leberman R. (1990). A second class of synthetase structure revealed by X-ray analysis of *Escherichia coli* seryl-tRNA synthetase at 2.5 Å. Nature.

[11] Ruff M., Krishnaswamy S., Boeglin M., Poterszman A., Mitschler A., Podjarny A., Rees B., Thierry J.C., Moras D. (1991). Class II Aminoacyl Transfer RNA Synthetases: Crystal Structure of Yeast Aspartyl-tRNA Synthetase Complexed with Trna (Asp). Science.

[12] Carter C.W. (2014). Urzymology: Experimental Access to a Key Transition in the Appearance of Enzymes. J. Biol. Chem..

[13] Li L., Francklyn C., Carter C.W. (2013). Aminoacylating Urzymes Challenge the RNA World Hypothesis. J. Biol. Chem..

[14] Li L., Weinreb V., Francklyn C., Carter C.W. (2011). Histidyl-tRNA Synthetase Urzymes: Class I and II Aminoacyl-tRNA Synthetase Urzymes have Comparable Catalytic Activities for Cognate Amino Acid Activation. J. Biol. Chem..

[15] Pham Y., Li L., Kim A., Erdogan O., Weinreb V., Butterfoss G., Kuhlman B., Carter C.W. (2007). A Minimal TrpRS Catalytic Domain Supports Sense/Antisense Ancestry of Class I and II Aminoacyl-tRNA Synthetases. Mol. Cell.

[16] Carter C.W., Duax W.L. (2002). Did tRNA Synthetase Classes Arise on Opposite Strands of the Same Gene?. Mol. Cell.

[17] Chandrasekaran S.N., Yardimci G., Erdogan O., Roach J.M., Carter C.W. (2013). Statistical Evaluation of the Rodin-Ohno Hypothesis: Sense/Antisense Coding of Ancestral Class I and II Aminoacyl-tRNA Synthetases. Mol. Biol. Evol..

[18] Martinez L., Jimenez-Rodriguez M., Gonzalez-Rivera K., Williams T., Li L., Weinreb V., Niranj Chandrasekaran S., Collier M., Ambroggio X., Kuhlman B., Erdogan O., Carter C.W. (2015). Functional Class I and II Amino Acid Activating Enzymes Can Be Coded by Opposite Strands of the Same Gene. J. Biol. Chem..

[19] Jimenez M., Williams T., González-Rivera A.K., Li L., Erdogan O., Carter C.W. (2014). Did Class 1 and Class 2 Aminoacyl-tRNA Synthetases Descend from Genetically Complementary, Catalytically Active ATP-Binding Motifs?. Biophys. J..

[20] Carter C.W., Li L., Weinreb V., Collier M., Gonzales-Rivera K., Jimenez-Rodriguez M., Erdogan O., Chandrasekharan S.N. (2014). The Rodin-Ohno Hypothesis That Two Enzyme Superfamilies Descended from One Ancestral Gene: An Unlikely Scenario for the Origins of Translation That Will Not Be Dismissed. Biol. Direct.

[21] Pham Y., Kuhlman B., Butterfoss G.L., Hu H., Weinreb V., Carter C.W. (2010). Tryptophanyl-tRNA synthetase Urzyme: A model to recapitulate molecular evolution and investigate intramolecular complementation. J. Biol. Chem..

[22] Schroeder G.K., Wolfenden R. (2007). The Rate Enhancement Produced by the Ribosome: An Improved Model. Biochemisty.

[23] Sievers A., Beringer M., Rodnina M.V., Wolfenden R. (2004). The ribosome as an entropy trap. Proc. Natl. Acad. Sci. USA.

[24] Rodin A., Rodin S.N., Carter C.W. (2009). On Primordial Sense-Antisense Coding. J. Mol. Evol..

[25] Weinreb V., Li L., Chandrasekaran S.N., Koehl P., Delarue M., Carter C.W. (2014). Enhanced Amino Acid Selection in Fully-Evolved Tryptophanyl-tRNA Synthetase, Relative to its Urzyme, Requires Domain Movement Sensed by the D1 Switch, a Remote, Dynamic Packing Motif. J. Biol. Chem..

[26] Li L., Carter C.W. (2013). Full Implementation of the Genetic Code by Tryptophanyl-tRNA Synthetase Requires Intermodular Coupling. J. Biol. Chem..

[27] Burbaum J., Schimmel P. (1991). Structural Relationships and the Classification of Aminoacyl-tRNA Synthetases. J. Biol. Chem..

[28] Burbaum J.J., Schimmel P. (1991). Assembly of a Class I tRNA Synthetase from Products of an Artificially Split Gene. Biochemtry.

[29] Burbaum J.J., Starzyk R.M., Schimmel P. (1990). Understanding Structural Relationships in Proteins of Unsolved Three-Dimensional Structure. Protein. Struct. Funct. Genet..

[30] Liu Y., Kuhlman B. (2006). RosettaDesign server for protein design. Nucleic Acids Res..

[31] Wolfenden R., Snider M.J. (2001). The Depth of Chemical Time and the Power of Enzymes as Catalysts. Acc. Chem. Res..

[32] Kirby A.J., Younas M. (1970). The Reactivity of Phosphate Esters. Reactions of Diesters with Nucleophiles. J. Chem. Soc. B Phys. Org..

[33] Stockbridge R.B., Wolfenden R. (2009). The Intrinsic Reactivity of ATP and the Catalytic Proficiencies of Kinases Acting on Glucose, N-Acetylgalactosamine, and Homeserine: A Thermodynamic Analysis. J. Biol. Chem..

[34] Kumar R.K., Yarus M. (2001). RNA-catalyzed amino acid activation. Biochemtry.

[35] Edgar R.C. (2004). MUSCLE: Multiple sequence alignment with high accuracy and high throughput. Nucleic Acids Res..

[36] Abascal F., Zardoya R., Posada D. (2005). ProtTest: Selection of best-fit models of protein evolution. Bioinformatics.

[37] Darriba D., Taboada G.L., Doallo R., Posada D. (2012). jModelTest 2: More models, new heuristics and parallel computing. Nat. Meth..

[38] Yang Z. (2007). PAML 4: A program package for phylogenetic analysis by maximum likelihood. Mol. Biol. Evol..

[39] Traut T.W. (2007). Allosteric Regulatory Enzymes.

[40] Traut T.W. (1986). Are proteins made of modules. Mol. Cell. Biochem..

[41] Peters J.W., Williams L.D. (2012). The Origin of Life: Look Up and Look Down. Astrobiology.

[42] Vestigian K., Woese C.R., Goldenfeld N. (2006). Collective Evolution and the Genetic Code. Proc. Natl. Acad. Sci. USA.

[43] Woese C.R. (1965). On the Origin of the Genetic Code. Proc. Natl. Acad. Sci. USA.

[44] Petrov A.S., Bernier C.R., Hsiao C., Norris A.M., Kovacs N.A., Waterbury C.C., Stepanov V.G., Harvey S.C., Fox G.E., Wartell R.M. (2014). Evolution of the Ribosome at Atomic Resolution. Proc. Natl. Acad. Sci. USA.

[45] Hsiao C., Lenz T.K., Peters J.K., Fang P.-Y., Schneider D.M., Anderson E.J., Preeprem T., Bowman J.C., O’Neill E.B., Lie L. (2013). Molecular paleontology: A biochemical model of the ancestral ribosome. Nucleic Acids Res..

[46] Noller H.F., Hoffarth V., Zimniak L. (1992). Unusual Resistance of Peptidyl Transferase to Protein Extraction Procedures. Science.

[47] Shakhnovich B.E., Dokholyan N.V., DeLisi C., Shacknovich E. (2003). Functional Fingerprints of Folds: Evidence for Correlated Structure-Function Evolution. J. Mol. Biol..

[48] Dokholyan N.V., Shakhnovich B., Shacknovich E.I. (2002). Expanding protein universe and its origin from the biological big bang. Proc. Natl. Acad. Sci. USA.

[49] Dokholyan N.V., Shakhnovich E.I. (2001). Understanding hierarchical protein evolution from first principles. J. Mol. Biol..

[50] Mullen G.P., Vaughn J.B., Mildvan A.S. (1993). Sequential Proton NMR Resonance Assignments, Circular Dichroism, and Structural Properties of a 50-Residue Substrate-Binding Peptide from DNA Polymerase I. Arch. Biochem. Biophys..

[51] Chuang W.-J., Abeygunawardana C., Pedersen P.L., Mildvan A.S. (1992). Two-Dimensional NMR, Circular Dichroism, and Fluorescence Studies of PP-50, a Synthetic ATP-Binding Peptide from the β-Subunit of Mitochondrial ATP Synthase. Biochem..

[52] Chuang W.-J., Abeygunawardana C., Gittis A.G., Pedersen P.L., Mildvan A.S. (1992). Solution Structure and Function in Trifluoroethanol of PP-50, an ATP-Binding Peptide from F_1_ATPase. Arch. Biochem. Biophys..

[53] Fry D.C., Byler D.M., Sisu H., Brown E.M., Kuby S.A., Mildvan A.S. (1988). Solution Structure of the 45-Residue MgATP-Binding Peptide of Adenylate Kinase As Examined by 2-d NMR, FTIR, and CD Spectroscopy. Biochem..

[54] Fry D.C., Kuby S.A., Mildvan A.S. (1985). NMR Studies of the MgATP Binding Site of Adenylate Kinase and of a 45-Residue Peptide Fragment of the Enzyme. Biochemtry.

[55] Schimmel P., Giegé R., Moras D., Yokoyama S. (1993). An operational RNA code for amino acids and possible relationship to genetic code. Proc. Natl. Acad. Sci. USA.

[56] Carter C.W., Wolfenden R. (2015). tRNA Acceptor-Stem and Anticodon Bases Form Independent Codes Related to Protein Folding. Proc. Natl. Acad. Sci. USA.

[57] Muñoz V., Serrano L. (1994). Intrinsic Secondary Structure Propensities of the Amino Acids, Using Statistical Φ-Ψ matrices: Comparison with Experimental Scales. Protein. Struct. Funct. Gen..

[58] Kramer R.M., Shende V.R., Motl N., Pace C.N., Scholtz J.M. (2014). Toward a Molecular Understanding of Protein Solubility: Increased Negative Surface Charge Correlates with Increased Solubility. Biophys. J..

[59] Franzen K.L., Kinsella J.E. (1976). Functional Properties of Succinylated and Acetylated Soy Protein. J. Agric. Food Chem..

[60] Szostak J. (2012). The eightfold path to non-enzymatic RNA replication. J. Syst. Chem..

[61] Glusker J.P., Katz A.K., Bock C.W. (1999). METAL IONS IN BIOLOGICAL SYSTEMS. Rigaku J..

[62] Andreini C., Bertini I., Cavallaro G., Holliday G.L., Thornton J.M. (2008). Metal ions in biological catalysis: From enzyme databases to general principles. J. Biol. Inorg. Chem..

[63] AbouHaidar M.G., Ivanovb I.G. (1999). Non-Enzymatic RNA Hydrolysis Promoted by the Combined Catalytic Activity of Buffers and Magnesium Ions. Z. Naturforsch. C.

[64] Henderson B.S., Schimmel P. (1997). RNA-RNA Interactions Between Oligonucleotide Substrates for Aminoacylation. Bioorg. Med. Chem..

[65] Achbergerová L., Nahálka J. (2011). Polyphosphate—an ancient energy source and active metabolic regulator. Microb. Cell Fact..

[66] Kornberg A. (1995). Inorganic Polyphosphate: Toward Making a Forgotten Polymer Unforgettable. J. Bact..

[67] Härtlein M., Cusack S. (1995). Structure, Function and Evolution of Seryl-tRNA Synthetases: Implications for the Evolution of Aminoacyl-tRNA Synthetases and the Genetic Code. J. Mol. Evol..

[68] Maizels N., Weiner A.M. (1994). Phylogeny from function: Evidence from the molecular fossil record that tRNA originated in replication, not translation. Proc. Natl. Acad. Sci. USA.

[69] Weiner A.M., Maizels N. (1987). tRNA-like structures tag the 3' ends of genomic RNA molecules for replication: Implications for the origin of protein synthesis. Proc. Natl. Acad. Sci. USA.

[70] Wächtershäuser G. (2014). The Place of RNA in the Origin and Early Evolution of the Genetic Machinery. Life.

[71] Wong J.T.-F. (2005). Coevolution theory of the genetic code at age thirty. BioEssays.

[72] Zhu T.F., Budin I., Szostak J.W. (2013). Vesicle Extrusion Through Polycarbonate Track-etched Membranes using a Hand-held Mini-extruder. Meth. Enzymol..

[73] Perona J.J., Gruic-Sovulj I. (2013). Synthetic and Editing Mechanisms of Aminoacyl-tRNA Synthetases. Top. Curr. Chem..

[74] Perona J.J., Hadd A. (2013). Structural Diversity and Protein Engineering of the Aminoacyl-tRNA Synthetases. Biochemistry.

[75] Bullock T., Uter N., Nissan T.A., Perona J.J. (2003). Amino Acid Discrimination by a class I aminoacyl-tRNA synthetase specified by negative determinants. J. Mol. Biol..

[76] Ghosh A., Sakaguchi R., Liu C., Vishveshwara S., Hou Y.-M. (2011). Allosteric Communication in Cysteinyl tRNA Synthetase A NETWORK OF DIRECT AND INDIRECT READOUT. J. Biol. Chem..

[77] Marquet R., lsel C., Ehresmann C., Ehresmann B. (1995). tRNAs as prirner of reverse transcriptases. Biochimie.

[78] Bayes T., Price R. (1763). An Essay towards solving a Problem in the Doctrine of Chance. By the late Rev. Mr. Bayes, F. R. S. communicated by Mr. Price, in a letter to John Canton, A. M. F. R. S.. Philos. Trans..

[79] Popper K. (1959). The Logic of Scientific Discovery.

[80] Yarus M. (2011). The meaning of a minuscule ribozyme. Phil. Trans. R. Soc. B.

[81] Turk R.M., Illangasekare M., Yarus M. (2011). Catalyzed and Spontaneous Reactions on Ribozyme Ribose. J. Am. Chem. Soc..

[82] Turk R.M., Chumachenkob N.V., Yarus M. (2010). Multiple translational products from a five-nucleotide ribozyme. Proc. Natl. Acad. Sci. USA.

[83] Yarus M. (2011). Life from an RNA World: The Ancestor within.

[84] Yarus M., Widmann J., Knight R. (2009). RNA-amino acid binding: A stereochemical era for the genetic code. J. Mol. Evol..

[85] Welch M., Majerfeld I., Yarus M. (1997). 23S rRNA Similarity from Selection for Peptidyl Transferase Mimicry. Biochemistry.

[86] Welch M., Chastang J., Yarus M. (1995). An Inhibitor of Ribosomal Peptidyl Transferase Using Transition-State Analogy. Biochemtry.

[87] Niwa N., Yamagishi Y., Murakami H., Suga H. (2009). A flexizyme that selectively charges amino acids activated by a water-friendly leaving group. Bioorg. Med. Chem. Lett..

[88] Sczepanski J.T., Joyce G.F. (2014). A cross-chiral RNA polymerase ribozyme. Nature.

[89] Lincoln T.A., Joyce G.F. (2009). Self-Sustained Replication of an RNA Enzyme. Science.

[90] Shechner D.M., Bartel D.P. (2011). The structural basis of RNA-catalyzed RNA polymerization. Nat. Struct. Mol. Biol..

[91] Johnston W.K., Unrau P.J., Lawrence M.S., Glasner M.E., Bartel D.P. (2001). RNA-Catalyzed RNA Polymerization: Accurate and General RNA-Templated Primer Extension. Science.

[92] Bartel D.P., Unrau P.J. (1999). Constructing an RNA world. Trends Biochem. Sci..

[93] Wochner A., Attwater J., Coulson A., Holliger P. (2011). Ribozyme-Catalyzed Transcription of an Active Ribozyme. Science.

[94] Noller H. (2004). The driving force for molecular evolution of translation. RNA.

[95] Ban N., Nissen P., Hansen J., Moore P., Steitz T.A. (2000). The Complete Atomic Structure of the Large Ribosomal Subunit at 2.4 Å Resolution. Science.

[96] Henkin T.M. (2009). RNA-dependent RNA switches in bacteria. Meth. Mol. Biol..

[97] Grundy F.J., Winkler W.C., Henkin T.M. (2002). tRNA-mediated transcription antitermination in vitro: Codon-anticodon pairing independent of the ribosome. Proc. Natl. Acad. Sci. USA.

[98] Baldwin R.L. (2012). Gas-liquid transfer data used to analyze hydrophobic hydration and find the nature of the Kauzmann-Tanford hydrophobic factor. Proc. Natl. Acad. Sci. USA.

[99] Kauzmann W. (1959). Some Factors in the Interpretation of Protein Denaturation. Adv. Protein Chem..

